# Application of a thermostable Baeyer–Villiger monooxygenase for the synthesis of branched polyester precursors

**DOI:** 10.1002/jctb.5623

**Published:** 2018-04-16

**Authors:** Marie AF Delgove, Matthew T Elford, Katrien V Bernaerts, Stefaan MA De Wildeman

**Affiliations:** ^1^ Maastricht University Aachen‐Maastricht Institute for Biobased Materials (AMIBM) Urmonderbaan The Netherlands

**Keywords:** multi‐enzymatic reactions, Baeyer–Villiger monooxygenase (BVMO), branched lactone synthesis, applied biocatalysis, polymer application, reaction engineering, space–time‐yield

## Abstract

**BACKGROUND:**

It is widely accepted that the poor thermostability of Baeyer–Villiger monooxygenases limits their use as biocatalysts for applied biocatalysis in industrial applications. The goal of this study was to investigate the biocatalytic oxidation of 3,3,5‐trimethylcyclohexanone using a thermostable cyclohexanone monooxygenase from Thermocrispum municipale (TmCHMO) for the synthesis of branched ϵ‐caprolactone derivatives as building blocks for tuned polymeric backbones. In this multi‐enzymatic reaction, the thermostable cyclohexanone monooxygenase was fused to a phosphite dehydrogenase (PTDH) in order to ensure co‐factor regeneration.

**RESULTS:**

Using reaction engineering, the reaction rate and product formation of the regio‐isomeric branched lactones were improved and the use of co‐solvents and the initial substrate load were investigated. Substrate inhibition and poor product solubility were overcome using continuous substrate feeding regimes, as well as a biphasic reaction system with toluene as water‐immiscible organic solvent. A maximum volumetric productivity, or space–time‐yield, of 1.20 g L^‐1^ h^‐1^ was achieved with continuous feeding of substrate using methanol as co‐solvent, while a maximum product concentration of 11.6 g L^‐1^ was achieved with toluene acting as a second phase and substrate reservoir.

**CONCLUSION:**

These improvements in key process metrics therefore demonstrate progress towards the up‐scaled Baeyer–Villiger monooxygenase‐biocatalyzed synthesis of the target building blocks for polymer application. © 2018 The Authors. *Journal of Chemical Technology & Biotechnology* published by John Wiley & Sons Ltd on behalf of Society of Chemical Industry.

## INTRODUCTION

Chemical Baeyer–Villiger oxidation of cyclic ketones is typically used for the synthesis of branched lactones – as polymeric building blocks for aliphatic polyesters – which has been the case for more than a century.^1^ This synthesis route has several disadvantages including limited regioselectivity, the use of toxic and usually halogenated oxidant species and solvents, and stoichiometric formation of unwanted by‐product,^2^ which leads to poor atom economy. Biocatalysis, particularly the use of Baeyer–Villiger monooxygenases (BVMOs), presents a greener alternative for lactone synthesis from cyclic ketone substrates.^3^ BVMOs have the advantage of using molecular oxygen as oxidant and forming water as by‐product. Moreover, these biocatalysts require milder reaction conditions, often operating at moderate temperatures and normal pressures. A major advantage of BVMOs is their regioselectivity, enabling the synthesis of the normal and abnormal lactones (i.e. insertion of one atom of oxygen at the most‐ or least‐substituted position, respectively). BVMOs display a broad substrate scope,^4^, ^5^ which include cyclic ketones with a heteroatom,^6^, ^7^ macrocyclic ketones,^8^, ^9^, ^10^, ^11^ and substituted derivatives of various ring size (for example, terpene‐based substrates^12^, ^13^).^14^, ^15^ BVMOs are also especially interesting in the synthesis of branched lactones, from which branched aliphatic polyesters with low glass transition temperatures can be made.^16^, ^17^, ^18^, ^19^, ^20^, ^21^, ^22^, ^23^, ^24^, ^25^

While the use of industrial biocatalysis is expanding into biotechnological processes for pharmaceutical building blocks,^26^, ^27^, ^28^ flavors and fragrances,^29^ and fine chemicals,^30^ BVMOs are not yet mainstream catalysts for the preparation of polymeric building blocks on an industrial scale. The potential of BVMOs has nonetheless been demonstrated with the oxidation of *rac‐*bicyclo[3.2.0]hept‐2‐en‐6‐one, using a cyclohexanone monooxygenase from *Acinetobacter calcoaceticus* NCIMB 9871 (AcCHMO; EC 1.14.13.22) in whole cells. The asymmetric oxidation of this key pharmaceutical intermediate has been performed at kg‐scale in pilot plant reactors.^31^, ^32^ Whole cells expressing AcCHMO, for example, reached a moderate productivity of 0.79 g L^‐1^ h^‐1^ for the synthesis of the monomer ϵ‐caprolactone.^33^ A cyclopentadecanone monooxygenase from *Pseudomonas* sp. strain HI‐70 (PsCPDMO; EC 1.14.13) has similarly been applied for the multi‐dozen gram scale preparation of an ϵ‐caprolactone derivative as a precursor for polyamides.^34^ These are to the best of our knowledge the most recent examples of the use of BVMOs for the synthesis of lactones at larger scale with application as building blocks for polymeric backbones.

So far, the main limitations to the application of BVMOs in industrial processes remain their limited robustness (i.e. low thermostability and poor solvent tolerance), substrate and product inhibition as well as the regeneration of their NAD(P)H co‐factor.^5^ Although AcCHMO is the most widely studied BVMO,^14^ it displays poor solvent tolerance as well as low thermostability which limits its use as a biocatalyst.^35^, ^36^ A robust cyclohexanone monooxygenase from *Thermocrispum municipale* DSM 44069 (TmCHMO; EC 1.14.13.22) has recently been discovered.^35^ This BVMO displays good thermostability (*T*_m_ = 48 °C) and very good solvent resistance, thus making it a good candidate for up‐scaled enzymatic oxidations. We recently reported on the broad substrate scope of TmCHMO with branched cyclic ketones including (+),(–)‐menthone, α,β‐thujone, jasmatone, and 3,3,5‐trimethylcyclohexanone.^12^ The products of the oxidation of 3,3,5‐trimethylcyclohexanone, which consist of a mixture of regio‐isomeric branched ϵ‐caprolactone derivatives, are of interest for the synthesis of aliphatic (co)polyesters.^37^, ^38^, ^39^

In order to use TmCHMO at large scale, it is necessary to ensure the regeneration of its NADPH co‐factor, since it is costly and prone to degradation.^40^ Phosphite dehydrogenase (PTDH) has the advantage of being able to use phosphite as a cheap, sacrificial substrate, while being thermostable and selective for NAD(P)^+^.^41^ Moreover, a self‐sufficient TmCHMO‐PTDH fused biocatalyst has been developed using genetic engineering.^35^, ^42^ In this work, our goal was to optimize the use of TmCHMO as biocatalyst for the oxidation of 3,3,5‐trimethylcyclohexanone for the synthesis of branched lactones as precursors for polymeric building blocks. For this, self‐sufficient enzymatic reactions were performed using TmCHMO‐PTDH with controlled reaction conditions (oxygen input, pH, and temperature) with a total volume of 30 mL. The effect of substrate loading, NADPH concentration, and biocatalyst loading were evaluated. A specific regioselective behavior was investigated, and compared with other BVMOs. The biocatalyzed oxidation was further optimized, using either a continuous substrate feeding strategy or a biphasic system, in order to increase achievable product concentrations and productivity.

## MATERIALS AND METHODS

### Chemicals

3,3,5‐trimethylcyclohexanone (98%, Sigma‐Aldrich), sodium phosphite dibasic pentahydrate (> 98%, Sigma Aldrich), dichloromethane (Biosolve), hexadecane (99.5%, TCI), acetonitrile (Biosolve), 1,4‐dioxane (Sigma Aldrich), methanol (Biosolve), ethanol (Biosolve), 1,3‐dioxolane (Acros organics), and ethyl acetate (Biosolve) were used as received. 3‐Chloroperbenzoic acid (70%, Sigma Aldrich) was stored at –4 °C. β‐Nicotinamide adenine dinucleotide phosphate reduced tetrasodium salt NADPH (95%, Alfa Aesar) and β‐nicotinamide adenine dinucleotide phosphate disodium salt NADP^+^ (97%, Alfa Aesar) were stored at –20 °C and solutions, in buffer, were prepared fresh prior to use.

### Biocatalyst production

TmCHMO‐PTDH, AcCHMO‐PTDH and RhCHMO‐PTDH were prepared as His‐PTDH‐fusion proteins in cell‐free extracts (CFE). *E. coli* NEB 10‐β cells transformed with the corresponding pCRE‐BVMO plasmid in Terrific Broth TB medium were preincubated overnight at 37 °C. The medium was then supplemented with ampicillin and *L*‐arabinose (0.02 %) and the cells were grown for 36 h at 24 °C. The culture medium to flask ratio was 1/10 and the culture medium was shaken at 135 rpm during fermentation. After growth, the cells were harvested by ultracentrifugation (4 °C, 6000 rpm, 15 min) and resuspended in TRIS/HCl buffer (50 mmol L^‐1^, pH 7.5). The cells were disrupted by sonication. Cell‐free extracts (CFE) were obtained after a centrifugation step (4 °C, 15000 rpm, 45 min) and were stored at –80 °C. The BVMO concentration in each CFE was estimated based on overexpression levels (evaluated from SDS‐PAGE, normalized by volume) and total protein concentrations (measured by Bradford assay).

### ^1^H and ^13^C NMR

Proton (^1^H) and carbon (^13^C) NMR spectra were recorded on a Bruker DPX‐300 MHz apparatus at ambient probe temperature in CDCl_3_. ^1^H NMR experiments were recorded with 32 scans and semi‐quantitative ^13^C NMR experiments were recorded with 1024 scans. Chemical shifts are reported in ppm.

### GC–MS

Gas chromatography coupled to mass spectrometry analyses were performed on a Shimadzu GC‐2010 Plus Gas Chromatography system equipped with a Shimadzu GC–MS‐QP2010 ultra mass spectrometer detector and a SH‐Rxi‐5 ms capillary column (30 m × 0.25 µm × 0.25 mm inner diameter). A mass/charge (m/z) range of 35–750 Da was analyzed. The temperature program was: starting temperature of 60 °C held for 2 min, temperature increased to 200 °C with a heating rate of 15 °C min^‐1^, and temperature finally increased to 320 °C at a heating rate of 20 °C min^‐1^. Between each temperature increase, a 2‐min hold time was implemented.

### GC‐FID

Gas chromatography analyses were performed using a Shimadzu GC‐2010 Plus Gas Chromatograph with a hydrogen flame‐ionization detector and a Supelco SPB‐1 capillary column (30 m × 0.25 µm × 0.25 mm inner diameter). The sample was injected at 250 °C (split ratio of 10, 2 µL injection volume). The temperature program was: starting temperature of 60 °C maintained for 2 min, temperature increased to 200 °C at a heating rate of 15 °C min^‐1^ and then maintained at 200 °C for 2 min, and temperature finally increased to 320 °C at a heating rate of 20 °C min^‐1^ and maintained at 320 °C for 2 min.

### Total protein concentration determination by Bradford assay

Samples containing 15 µL of CFE (diluted 1/100 v v^‐1^ in TRIS/HCl buffer at 50 mmol L^‐1^ and pH 7.5) and 150 µL of Bradford reagent (Bio‐Rad) were prepared in triplicate. Absorbance was measured at 595 nm in a 96‐well microtiter plate with a Multiskan GO microplate spectrophotometer at 22 °C. A calibration curve was made with bovine serum albumin (Sigma Aldrich). The total protein concentration of the CFE was determined as 13.2 ± 2.0 mg mL^‐1^ (TmCHMO‐PTDH), 12.5 ± 1.4 mg mL^‐1^ (AcCHMO‐PTDH), and 14.2 ± 1.2 mg mL^‐1^ (RhCHMO‐PTDH).

### SDS‐PAGE

Sodium dodecyl‐polyacrylamide gel electrophoresis was performed with the cell‐free extract after sonication and each supernatant and resuspended pellet after ultracentrifugation following sonication (10 µL samples). The samples were incubated at 95 °C for 5 min after addition of SDS loading dye. After centrifugation, the samples were loaded onto an SDS‐PAGE Gel (GenScript, USA) with a protein ladder of 10 to 200 kDa (PageRuler, pre‐stained Thermofischer). The gels were run at 120 V on a Mini‐PROTEAN® Tetra Vertical Electrophoresis Cell (Bio‐Rad). After the run, the gel was rinsed with water and stained with Coomassie InstantBlue™. The overexpression levels were estimated to 10–20% for TmCHMO‐PTDH, 20–30% for AcCHMO‐PTDH and 10–20% for RhCHMO‐PTDH.

### Determination of the concentration of active BVMO by spectrophotometry

The active BVMO concentration was measured using a flavin‐reduction method.^43^ The absorbance spectrum of the BVMOs in their oxidized form (CFE diluted 1/10 in TRIS/HCl buffer at 50 mmol L^‐1^ and pH 7.5) was measured from 300 to 700 nm with a JASCO V‐330 spectrophotometer. NADPH (500 µmol L^‐1^) was added and the samples were incubated at room temperature for 10 min to afford the BVMOs in their reduced form. The absorbance spectrum of the BVMOs was measured again. The concentration of active BVMO was determined as [BVMO] = (*A*_440, ox_ ‐ *A*_440, red_) / ϵ_440, diff_ with ϵ_440, diff_ = 10.15 mM^‐1^ cm^‐1^. The concentrations in the CFE were determined as [TmCHMO‐PTDH] = 37 µmol L^‐1^, [AcCHMO‐PTDH] = 96 µmol L^‐1^, and [RhCHMO‐PTDH] = 74 µmol L^‐1^.

### Bioreactor set‐up

Biocatalyzed reactions were performed with a Metrohm 877 Titrino plus titration apparatus. The reactor was a double‐walled vessel with a volume of 90 mL whose temperature was kept constant at 30 °C. Water‐saturated air was bubbled through the reaction mixture at a rate of 8 mL min^‐1^ with a Vögtlin red‐y flow controller. The pH was maintained at 8.0 by automatic titration of NaOH (1 mol L^‐1^, Merck).

### Determination of the kinetics for biocatalyzed reactions using GC‐FID

Aliquots of the reaction mixture (50 µL) were taken and mixed with acetonitrile (950 µL) containing hexadecane at a concentration of 100 µmol L^‐1^ as external standard. The sample was centrifuged, using an Eppendorf centrifuge 5424, to remove precipitated protein and analyzed by GC‐FID. Concentration of substrate and lactones were determined using calibration curves.

### Typical biocatalyzed reactions with single batch addition

The reaction vessel was loaded with 250 µmol L^‐1^ NADP^+^, 2–50 mmol L^‐1^ of 3,3,5‐trimethylcyclohexanone (8.4 to 210.33 mg), phosphite (125 mmol L^‐1^), and 10% v v^‐1^ co‐solvent. The stock solutions were prepared in a KPi buffer at 25 mmol L^‐1^ at pH 8.0. The reaction mixture was stirred at 500 rpm and air was bubbled through at a rate of 8 mL min^‐1^. The TmCHMO‐PTDH CFE was added to give an enzyme concentration of 4 µmol L^‐1^ (3 mL, 10% v v^‐1^) to start the reaction. Kinetics were followed by GC‐FID analysis.

### Typical biocatalyzed reactions with continuous substrate feeding (CSF)

The reaction vessel was loaded with 250 µmol L^‐1^ NADP^+^, phosphite (125 mmol L^‐1^) and 10% v v^‐1^ methanol, in KPi buffer at 25 mmol L^‐1^ at pH 8.0. The reaction mixture was stirred at 500 rpm and air was bubbled through at a rate of 8 mL min^‐1^. The TmCHMO‐PTDH CFE was added to give an enzyme concentration of 4 µmol L^‐1^ (3 mL, 10% v v^‐1^). Pure 3,3,5‐trimethylcyclo hexanone was fed continuously using a syringe pump (KDS Legato 110) with a feeding rate of 10 or 20 mmol L^‐1^ h^‐1^ (42.1 or 84.1 mg h^‐1^) for 2, 3 or 5 h. Kinetics were followed by GC‐FID analysis.

### Typical biocatalyzed reactions with water/toluene biphasic system

The reaction vessel was loaded with 250 µmol L^‐1^ NADP^+^, and phosphite (125 mmol L^‐1^) in KPi buffer at 25 mmol L^‐1^ at pH 8.0 (volume of the aqueous phase 30 mL) and stirred at 500 rpm. 3,3,5‐Trimethylcyclohexanone (210.3 mg) was dissolved in toluene (15 mL) to give a solution of 100 mmol L^‐1^ substrate in the toluene phase (33% v v^‐1^). The solution was added to the reaction medium. Air was bubbled through the reaction medium at a rate of 8 mL min^‐1^. The TmCHMO‐PTDH CFE was added (3 mL, 10% v v^‐1^, final concentration 4 µmol L^‐1^) to start the reaction. Kinetics were followed by GC‐FID analysis. Stirring was stopped prior to each sampling point in order to achieve phase separation and take a sample from both the aqueous and the toluene phase. The reaction was stopped after 24 h.

### Product recovery procedure of the biocatalyzed reactions

Reaction mixtures consisting of one phase were centrifuged to separate precipitated protein. The supernatant solution was extracted with ethyl acetate (3 × 30 mL). The combined organic layers were dried over MgSO_4_. The solvent was removed under rotary evaporation to afford a colorless to light‐yellow liquid (110 mg, 47% yield). In the case of the biphasic system, the phases were centrifuged separately to remove precipitated protein. The solvent in the toluene phase was removed in vacuo to afford a colorless to light yellow liquid (88.8 mg, 38% yield). The aqueous phase was extracted with ethyl acetate (3 × 30 mL). The solutions were dried over MgSO_4_ and ethyl acetate was removed under rotary evaporation to afford a colorless to light‐yellow liquid (11.1 mg, 5% yield). Regio‐isomeric lactones β,β,δ‐trimethyl‐ϵ‐caprolactone **1a** and β,δ,δ‐trimethyl‐ϵ‐caprolactone **1b.** GC–MS (m/z): **1a** (C_9_H_16_O_2_), 126, 111, 108, 93, 83, 69, 56, 41, 39; **1b** (C_9_H_16_O_2_), 156, 126, 111, 99, 83, 69, 56, 41, 39. ^1^H NMR (300 MHz, CDCl_3_): δ (ppm) = 0.85 (s, 6H); 0.94 (s, 6H); 0.98 (s, 6H); 1.04‐1.28 (m, 2H); 1.47–1.63 (m, 2H); 1.82–2.12 (m, 2H); 2.25–2.47 (m, 3H); 2.61–2.72 (m, 1H); 3.64–3.74 (m, 1H); 3.80–4.04 (m, 3H).^13^C NMR (300 MHz, CDCl_3_): δ (ppm) = 17.9, 21.2, 23.0, 23.8, 25.3, 27.2, 29.6, 30.4, 32.3, 33.1, 40.9, 45.7, 50.5, 50.8, 73.3, 75.6, 173.0, 173.7.

### Determination of the stability of TmCHMO‐PTDH to toluene as second phase

TmCHMO‐PTDH (250 µL of CFE) was incubated at 30 °C for 24 h in a reaction mixture consisting of 33% v v^‐1^ toluene (1.25 mL) in KPi buffer (2.25 mL) at 25 mmol L^‐1^ and pH 8.0. The remaining activity was measured by spectrophotometric assay in a 96‐well plate with a Multiskan GO microplate spectrophotometer at 30 °C. The NADPH consumption of mixtures containing sample from the incubated enzyme (20 µL), 500 µmol L^‐1^ NADPH (160 µL) and 50 mmol L^‐1^ cyclohexanone (20 µL) was measured at 340 nm. The remaining activity was measured relative to the initial *k*_obs_ calculated as *k*_obs_ = (d*A*_340_/dt) / ([TmCHMO‐PTDH] × ϵ_340_ × *ℓ*) with *A* absorbance, ϵ_340_ extinction coefficient of NADPH at 340 nm (6.22 mM^‐1^ cm^‐1^), [TmCHMO‐PTDH] = 37 µmol L^‐1^, and *ℓ* = 0.5 cm.

### Solubility of 3,3,5‐trimethylcyclohexanone 1 and lactone mixture 1a and 1b in organic solvents

The solubility of the substrate and products was assessed by dissolving 50 mg in 1 mL of solvent (357 mmol L^‐1^ for **1**, 321 mmol L^‐1^ for the mixture of **1a** and **1b**) at room temperature. The solubility was assessed after 24 h by visual inspection. The solvents tested were acetic acid, acetone, acetonitrile, benzonitrile, benzyl alcohol, 2‐butanol, γ‐butyrolactone, chlorobenzene, chloroform, *m*‐cresol, cyclohexane, cyclohexanone, di‐*n*‐butyl ether, diethyl amine, diethylene glycol, *N*‐*N*‐dimethyl acetamide, dimethyl formamide, dimethyl sulfoxide, ethanol, ethyl acetate, ethyl benzene, ethylene glycol, ethylene glycol monomethyl ether, formamide, hexane, isobutyl alcohol, methanol, methyl ethyl ketone, *N*‐methyl‐2‐pyrrolidone, dichloromethane, nitrobenzene, 1‐octanol, 1‐pentanol, 1‐propanol, propylene carbonate, 1‐methoxy‐2‐propanol, tetrahydrofuran, toluene, valeronitrile, water, *N*‐methyl formamide, 2‐phenyl ethanol, 1,3‐dioxolane, and 1,4‐dioxane.

### Stability of 3,3,5‐trimethylcyclohexanone 1 and lactone mixture 1a and 1b in typical reaction medium

Two reaction mixtures containing either 50 mmol L^‐1^ of 3,3,5‐trimethylcyclohexanone or the mixture of lactones ‐ as synthesized by chemical Baeyer–Villiger oxidation using 3‐chloroperbenzoic acid as the oxidant‐^38^ 10% v v^‐1^ 1,4‐dioxane, 100 µmol L^‐1^ of NADPH, 125 mmol L^‐1^ of phosphite were prepared. The medium was prepared with KPi buffer (25 mmol L^‐1^) at pH 8.0. The reaction mixtures were left to incubate at 30 °C in a flask shaking apparatus. The stability of the substrate and of the product were evaluated by measuring the remaining amount by GC‐FID. Samples were prepared by diluting aliquots of the reaction mixture in acetonitrile (1/1 v v^‐1^).

### Kinetics of the chemical Baeyer–Villiger oxidation of 3,3,5‐trimethylcyclohexanone

3,3,5‐Trimethylcyclohexanone (0.56 g, 4 mmol, 1 eq) was dissolved in dichloromethane (200 mL) to a concentration of 20 mM. 3‐Chloroperbenzoic acid (1.79 g, 8 mmol, 2 eq) was added in one batch. The solution was stirred at room temperature for 28 days. Kinetics were followed by taking aliquots of the reaction mixture (1 mL) and were directly analyzed by GC‐FID.

## RESULTS AND DISCUSSION

**Scheme 1:**
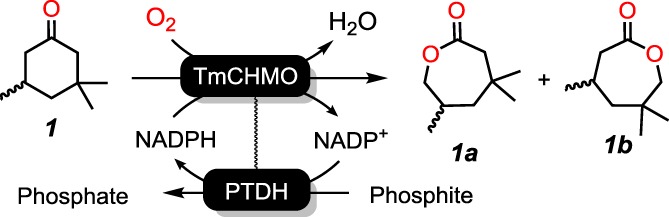
Biocatalyzed oxidation of 3,3,5‐trimethylcyclohexanone **1** to distal lactone **1a** and proximal lactone **1b** using a self‐sufficient TmCHMO‐PTDH fused biocatalyst.

Two regio‐isomeric lactones can be obtained from the biocatalyzed oxidation of the chosen substrate, 3,3,5‐trimethylcyclohexanone **1**, namely: the distal lactone β,β,δ‐trimethyl‐ϵ‐caprolactone **1a** (least‐substituted δ‐position), and the proximal lactone β,δ,δ‐trimethyl‐ϵ‐caprolactone **1b** (most‐substituted δ‐position) (Scheme 1). It should be noted that no resolution of the racemic mixure of the substrate **1** was observed since both enantiomers were converted by TmCHMO with conversions higher than 50%. Additionally, chirality of the monomers is not expected to be critical for polymerization for the targeted applications.

### Characterization of the biocatalyzed oxidation

In order to investigate the best reaction conditions to perform enzymatic oxidations in a 30 mL reaction medium, several parameters and their effects were investigated, namely the substrate loading, possible oxygen limitations via biocatalyst loading experiments, the effect of the co‐solvent on the reaction rate, and the concentration of NADP^+^. Possible substrate inhibition effects were investigated by performing biocatalysis reactions with an increasing initial loading of the substrate **1** from 2 to 75 mmol L^‐1^, using 10% v v^‐1^ acetonitrile to solubilize the substrate and the formed products (Table 1). This solvent was chosen given the very robust solvent resistance of TmCHMO to acetonitrile (79% residual activity after 20 h in 14% v v^‐1^ acetonitrile at 20 °C).^12^

**Table 1:** Effect of substrate concentration on conversion, specific activity, product concentration, yield, and space time yield (STY). Reaction conditions: [substrate] = 2–75 mmol L^‐1^, 10% v v^‐1^ acetonitrile, [TmCHMO‐PTDH] = 4 µmol L^‐1^, [NADP^+^] = 250 µmol L^‐1^

[sub]_i_ a (mmol L^‐1^)	Time (h)	Conv^a^ (%)	Specific activity^b^ (µmol min^‐1^ µmol_E_^‐1^)	[prod]_exp_ a (mol L^‐1^)	Yield (%)	STY_th_ c (g L^‐1^ h^‐1^)	STY_exp_ d (g L^‐1^ h^‐1^)
2	0.5	100	20.4	1.2	> 50	0.65	0.37
6	1.5	100	21.7	5.9	> 95	0.60	0.61
8	2.5	100	17.7	7.6	> 95	0.49	0.47
18	23.5	94	21.2	14.6	95	0.18	0.10
50	24	86	n/a	5.9	12	0.30	0.04
75	24	85	n/a	6.6	9	0.49	0.04

a Conversion and final product concentration calculated from GC–FID analysis with calibration curves, with conversion = (1 – ([sub]_final_/[sub]_initial_)) × 100.

b Amount of substrate converted per min per amount of enzyme (µmol_Substrate_ min^‐1^ µmol^‐1^_Enzyme_) calculated with linear regression on the initial reaction rate by plotting the substrate concentration *versus* time with [TmCHMO‐PTDH] = 4 µmol L^‐1^.

c Theoretical space time yield STY_th_ = [substrate]_initial_ × M_s_ / (V × t) with M_s_ molar mass of 3,3,5‐trimethylcyclohexanone = 140.22 g mol^‐1^, V volume of the reactor = 30 mL and t the total reaction time.

d Experimental STY_exp_ = [product]_final_ × M_p_ / (V × t) with M_p_ molar mass of the products ***1a*** and ***1b*** = 156.22 g mol^‐1^, V volume of the reactor = 30 mL and t the total reaction time.

**Figure 1:**
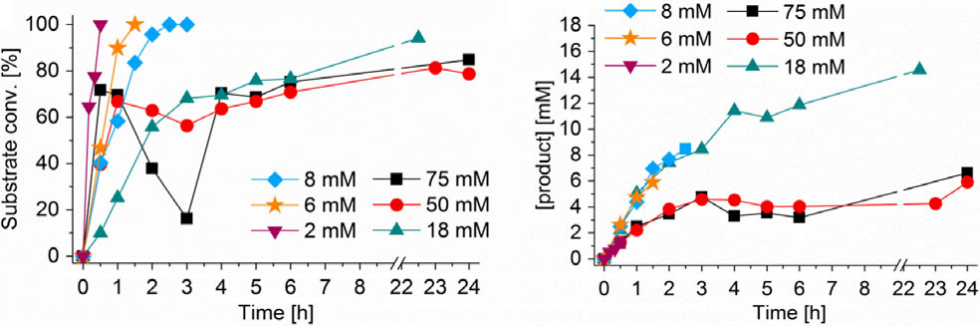
Kinetics of the biocatalyzed reactions with decreasing targeted substrate concentration with (a) substrate conversion vs time; and (b) product concentration vs time. Reaction conditions: [substrate] = 2–75 mmol L^‐1^, 10% v v^‐1^ acetonitrile, [TmCHMO‐PTDH] = 4 µmol L^‐1^, [NADP^+^] = 250 µmol L^‐1^.

Up to 8 mmol L^‐1^ substrate loading, full substrate conversion was obtained within 2.5 h, with very good mass balance and in solution yield (> 95%), except for the 2 mmol L^‐1^ substrate loading which seems to be the limiting lower concentration for analysis. Although an initial substrate loading of about 18 mmol L^‐1^ resulted in the highest in solution product concentration (2.3 g L^‐1^), the corresponding STY was impacted by the long reaction time required for conversion of the substrate (Fig. 1(a)). For substrate concentrations higher than 18 mmol L^‐1^, the reaction time increased as a result of substrate inhibition. Although conversions higher than 85% were observed, the product concentrations remained low and were accompanied by a poor mass balance (Fig. 1(b), Table 1).

To investigate whether the poor mass balance could be attributed to degradation of the substrate or products, their stability was analyzed in buffer and in the absence of biocatalyst, where no instability was observed (Figure S1, Supporting information). The loss of product in the aqueous phase was therefore attributed to poor water solubility of the lactones for concentrations higher than 18 mmol L^‐1^. Additionally, the specific activity is similar for a substrate concentration between 2 and 18 mmol L^‐1^, which indicates that no substrate inhibition can be observed for substrate loadings lower than 18 mmol L^‐1^ (Table 1). For further investigation of the optimum reaction conditions a substrate concentration of 10 mmol L^‐1^ was thus chosen (see Figure S2 for kinetics).

Stoichiometric oxygen is required to oxidize the ketone **1** to the lactone products **1a** and **1b**. To that end, possible oxygen limitations were evaluated by changing the biocatalyst loading. In the case of a reaction which is not oxygen‐limited, increasing the biocatalyst loading should result in an increased reaction rate. However, when oxygen is the limiting reactant, the addition of more biocatalyst will not increase the reaction rate. In order to compare the rates at various biocatalyst loadings, the substrate conversions were plotted against a normalized x‐axis, taking into account the enzyme concentration (Figure S3). This biocatalyst loading series clearly indicates that no oxygen limitation occurs because the specific activity was similar for decreasing biocatalyst concentration, although a small loss of activity was observed for the lowest biocatalyst concentration ([TmCHMO‐PTDH] = 0.64 µmol L^‐1^). As such, a concentration of 4 µmol L^‐1^ of biocatalyst was thus selected for further experiments.

**Figure 2:**
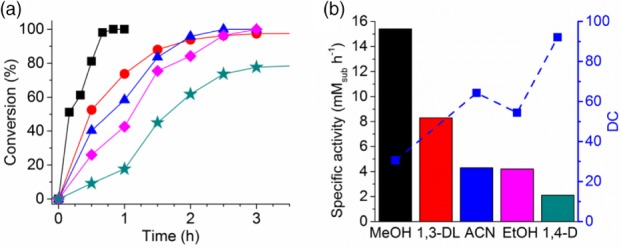
Effect of co‐solvent (10% v v^‐1^) on bioconversions with (a) substrate conversion as a function of time, and (b) specific activity of substrate (mM_sub_ h^‐1^) shown as bars on the left y‐axis, and denaturation capacity (DC) of the co‐solvents (blue squares with dotted line) on the y‐right axis. The specific activity of substrate conversion was calculated by linear regression on the initial kinetics of the biotransformations (first 40 min for methanol, 60 min for 1,3‐dioxolane, and 90 min for other co‐solvents), with MeOH, methanol (black); 1,3‐DL, 1,3‐dioxolane (red); ACN, acetonitrile (blue); EtOH, ethanol (pink); and 1,4‐D, 1,4‐dioxane (green). Reaction performed with [substrate] = 10 mmol L^‐1^, 10% v v^‐1^ co‐solvent, [TmCHMO‐PTDH] = 4 µmol L^‐1^, [NADP^+^] = 250 µmol L^‐1^ and [phosphite] = 125 mmol L^‐1^ in KPi buffer (25 mmol L^‐1^) at pH 8, 30 °C, 8 mL min^‐1^ airflow. Denaturation capacities values from reference 44.

The use of co‐solvent is required to solubilize the substrate and products, given their hydrophobic character (log *P* = 2.56 for **1,** 2.14 and 2.20 for **1a** and **1b** respectively, *P* partition coefficient, calculated as log *P*_oct/wat_ = log ([substrate]_octanol_ / [substrate]_un‐ionized water_) according to ChemDraw Professional 15.1). The substrate and products are fully miscible in many organic solvents at a concentration up to 50 g L^‐1^ (about 300 mmol L^‐1^). The effect of a series of water‐miscible co‐solvents at 10% v v^‐1^ on the conversion was evaluated (Fig. 2(a)). The substrate and product concentrations of biocatalyzed oxidations with different co‐solvents as a function of time are given in Figure S4a–f. Methanol displayed the highest specific activity, which is consistent with its low denaturation capacity (DC) (Fig. 2(b)).^44^ Conversely, 1,4‐dioxane, which has the highest DC of all the co‐solvents studied, slowed the reaction rate by a factor of 7. Both acetonitrile and ethanol had similar effects on the reaction rate. Using methanol allowed full substrate conversion within 1 h, whereas closer to 3 h was required for 1,3‐dioxolane, acetonitrile, or ethanol. Overall, a clear correlation could be established between the DC of the co‐solvents and their effect on the specific activity (Fig. 2(b)).

Decreasing the NADP^+^ concentration from 250 µmol L^‐1^ to 50 µmol L^‐1^ and 100 µmol L^‐1^ resulted in slower kinetics (Figure S5a‐b). More importantly, the final conversions were very similar, independent of the NADP^+^ concentration. The faster kinetics at higher co‐factor concentration may be due to improved enzyme stability. The half‐life of some BVMOs, including AcCHMO, has been shown to increase in the presence of larger available amount of NADPH.^45^ In the case of our study, NADPH is generated *in situ* by the PTDH which is fused to the BVMOs. For this reason, a NADP^+^ concentration of 250 µmol L^‐1^ was chosen for further investigation of the biocatalyzed oxidation.

### Influence on ratio of regio‐isomeric lactones

**Figure 3:**
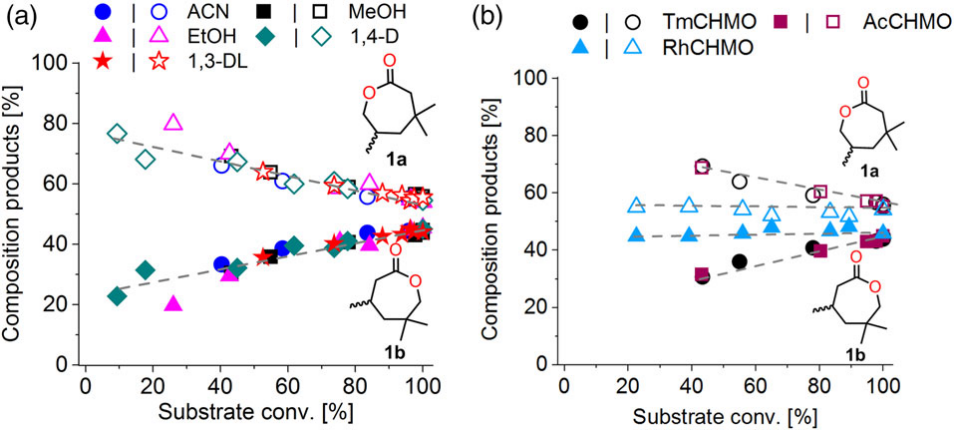
Composition of lactone products (%) as a function of substrate conversion for biocatalyzed reactions (a) with TmCHMO‐PTDH in the presence of 10% v v^‐1^ co‐solvent with methanol (MeOH, black squares), 1,3‐dioxolane (1,3‐DL, red stars), acetonitrile (ACN, blue circles), ethanol (EtOH, pink triangles), and 1,4‐dioxane (1,4‐D, green diamonds), and (b) with TmCHMO‐PTDH (black circles), AcCHMO‐PTDH (purple squares) and RhCHMO‐PTDH (blue triangles) in the presence of 10% v v^‐1^ methanol. Empty symbols indicate the distal lactone **1a** and full symbols indicate the proximal lactone **1b**. Reactions conditions: [substrate] = 10 mmol L^‐1^, 10% v v^‐1^ BVMO cell free extract (CFE), [NADP^+^] = 250 µmol L^‐1^.

The ratio of regio‐isomers resulting from the oxidation of 3,3,5‐trimethylcyclohexanone by TmCHMO‐PTDH was found to change considerably during the biocatalyzed reaction. At low substrate conversion, the formation of the distal lactone **1a** was favored (up to 75:25 **1a**:**1b** at 10% conversion) while at full substrate conversion, the ratio equilibrated to 55:45 (**1a**:**1b**). This phenomenon was found to be co‐solvent independent for the range of co‐solvents evaluated, which included alcohols, ethers and nitrile (Fig. 3(a)). A similar change in the ratio of the regio‐isomers during the course of the reaction was also observed when using AcCHMO, whereas it remained stable when the reaction was catalyzed by a cyclohexanone monooxygenase from *Rhodococcus* sp. HI‐70 (RhCHMO; 1.14.13.22), both enzymes being fused to PTDH (Fig. 3(b)). Interestingly, the formation of each regio‐isomer was linear over time, independent of both the co‐solvent and the enzyme (Figure S4a‐f and Figure S6a‐b). The rate of formation of the distal lactone **1a** was initially faster than that of the proximal lactone **1b**. However, both regio‐isomeric lactones were produced from the start of the reaction. It is therefore unlikely that the change in the ratio of regio‐isomers is due to product inhibition induced by one of the lactones. Moreover, the ratio of lactone formation seems to be independent of the substrate and product concentration. The same pattern was indeed observed when comparing TmCHMO‐PTDH catalyzed oxidations with different substrate supply, namely initial substrate loading and continuous substrate feeding (CSF), in which the ketone concentration remained very low throughout the reaction (Figure S7a‐b). This change in the ratio of regio‐isomeric products over time is thus enzyme dependent and co‐solvent independent (at least for TmCHMO‐PTDH).

In order to verify that this phenomenon was not intrinsic to Baeyer–Villiger oxidations, the ratio of regio‐isomeric products formed during a chemical Baeyer–Villiger oxidation of **1** was studied, using a comparable substrate concentration (i.e. 20 mmol L^‐1^) (Figure S8). Interestingly, the chemical Baeyer–Villiger was found to favor the formation of the proximal lactone **1b,** while the BVMOs favored the formation of the distal lactone **1a** (55:45 and 45:55 **1b**:**1a**, respectively). Unlike its biocatalyst counterpart, the chemical Baeyer–Villiger oxidation does not show any clear correlation between substrate conversion and ratio of regio‐isomeric products formed, at least under the present experimental conditions.^2^

### Strategies to improve the process metrics of the biocatalyzed oxidations

As shown previously, the biocatalyzed oxidation suffers from substrate inhibition at high substrate loading while low substrate concentrations afford lower product concentrations (Table 1). Consequently, strategies to increase the substrate and product concentration while maintaining a reduced reaction time were investigated in order to improve the productivity of TmCHMO catalyzed oxidations. These strategies include continuous substrate feeding (CSF) on the one hand and the use of an organic‐aqueous biphasic catalytic system on the other hand. In the case of CSF, the substrate concentration is maintained low throughout the reaction by gradual addition of the substrate while allowing for product accumulation. In the case of the biphasic reactions, in which an immiscible organic solvent forms as second phase in which both the substrate and the product are soluble, the organic layer acts as a substrate reservoir and product sink. Both strategies aim at avoiding substrate inhibition while maintaining a high product concentration, and are typical strategies applied for bioprocess intensification.^46^

**Figure 4:**
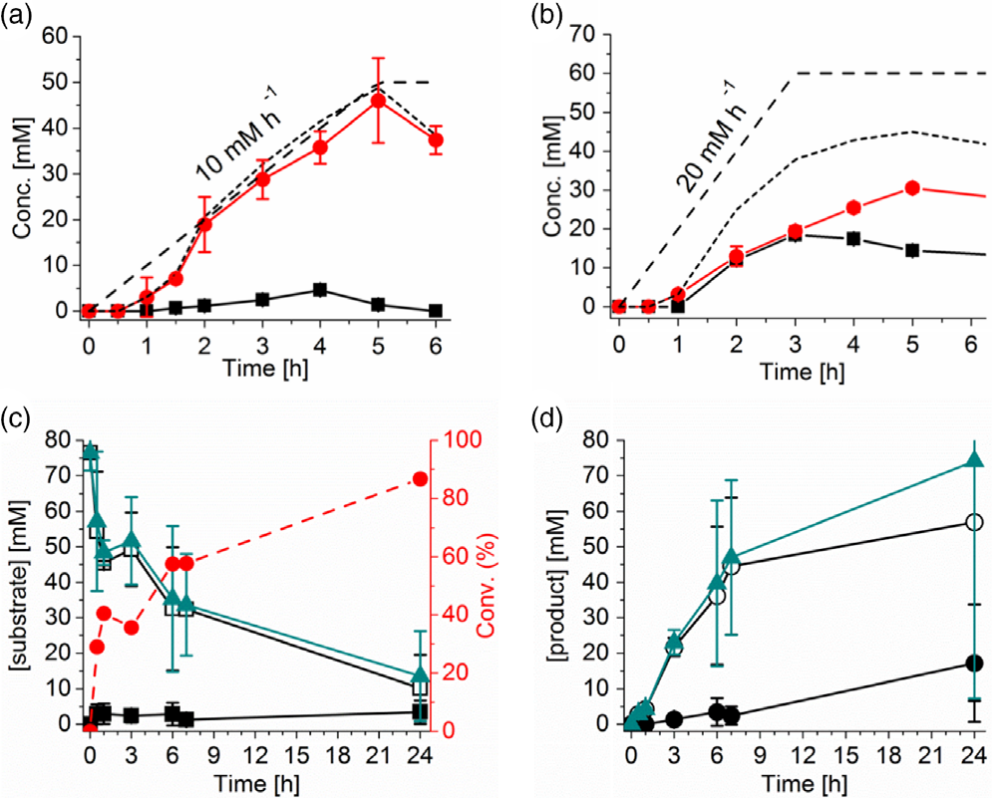
Biocatalyzed reactions with continuous substrate feeding (CSF) showing concentration of substrate (black squares) and product (red circles) as a function of time with a feed rate of (a) 10 mmol L^‐1^ h^‐1^ over 5 h, (b) 20 mmol L^‐1^ h^‐1^ over 3 h. Black dashed lines indicate the substrate feeding rate, and black dotted lines indicate the sum of substrate and product concentrations. Reactions conditions: 10% v v^‐1^ methanol, [TmCHMO‐PTDH] = 4 µmol L^‐1^, [NADP^+^] = 250 µmol L^‐1^. Below: biphasic biocatalyzed oxidation with toluene with (c) substrate concentration and substrate conversion (red dashed line), and (d) production concentration as a function of time. Concentrations in the toluene phase are shown with empty symbols, concentrations in the water phase with black symbols and the sum of the concentrations with green triangles. Reactions conditions: [substrate] = 100 mmol L^‐1^ in toluene (33% v v^‐1^), [TmCHMO‐PTDH] = 4 µmol L^‐1^, [NADP^+^] = 250 µmol L^‐1^.

Continuous substrate feeding (CSF) enzymatic reactions with feeding rates of 10 and 20 mmol L^‐1^ h^‐1^ were performed. Methanol was chosen as co‐solvent to allow for sufficient product solubility, since this co‐solvent was shown to give the highest specific activity (Fig. 2). Additionally, TmCHMO is particulary tolerant to methanol since this biocatalyst can retain 96% of conversion yield in the presence of 30% v v^‐1^ methanol for the conversion of 2‐butanone.^35^ While the substrate was almost instantaneously converted when fed into the reaction mixture at a rate of 10 mmol L^‐1^ h^‐1^, substrate accumulation was observed when the feeding rate was doubled to 20 mmol L^‐1^ h^‐1^ (Fig. 4(a) and 4(b)).

Additionally, product concentration followed the substrate feed in the first case, indicating immediate oxidation of the substrate. However, this was not the case when the substrate feed was doubled, although the substrate concentration decreased as soon as the feed of substrate was stopped. This increase of conversion after feeding of the substrate has stopped indicates that the ketone accumulation throughout the first 3 h of the reaction was due to the substrate feeding rate being above the maximum volumetric biocatalyst activity. An enzyme‐catalyzed reaction containing an initial product concentration of 40 mmol L^‐1^ indicated that product inhibition occurs for product concentrations between 20 mmol L^‐1^ and 40 mmol L^‐1^ since substrate conversion did not follow substrate feed and did not result in further product formation (Figure S9).

One strategy to prevent product inhibition is to ensure that the product concentration remains low in the aqueous phase, where the biocatalyst is present. A biphasic system was therefore implemented to increase substrate loading and avoid substrate and/or product inhibition. The tolerance of TmCHMO‐PTDH to toluene was studied by measuring the remaining activity of the biocatalyst in the presence of toluene. It was shown that 33% v v^‐1^ toluene resulted in a similar remaining enzyme activity compared to in the presence of buffer only (Figure S10). A biocatalyzed reaction with 33% v v^‐1^ toluene as second phase was performed, i.e. with 30 mL of aqueous phase and 15 mL of toluene. The concentration of the substrate and products were measured in both phases (Fig. 4(c) and 4(d)). Although the majority of the substrate and products were located in the toluene phase, a non‐negligible amount was also measured in the aqueous phase. Partitioning of the ketone and lactones was therefore not as straightforward as expected, despite what the log *P* values would suggest (log *P* toluene = 2.52), which complicated product recovery. Of all the strategies evaluated, including CSF and a biphasic system with toluene as second phase, the latter resulted in the highest product concentration (11.6 g L^‐1^ for both phases), due to the high initial substrate loading of 100 mmol L^‐1^ in the toluene phase. High conversion could only be obtained after 24 h, which resulted in a decreased productivity (0.48 g L^‐1^ h^‐1^) (Table 2). This is probably the result of the protein denaturation observed because of the increased interphase between the organic and aqueous phase, which decreased the amount of active biocatalyst. Additionally, this denatured protein present at the interphase is suspected to be a reservoir for non‐isolated substrate and product during normal product recovery procedures.

**Table 2:** Comparison of the process metrics of the best performing biocatalyzed oxidations

Entry	Reaction type	Solvent	[sub]_i_ a (g L^‐1^)	Time (h)	Conv.^b^ (%)	[prod]_exp_ b (g L^‐1^)	Biocat yield^c^ (g_prod_/g_enz_)	STY_exp_ d (g L^‐1^ h^‐1^)	TON^e^	Isolated yield (%)
1	Batch	ACN	1.1	2.5	100	1.2	4.3	0.46	18 375	67
2	Batch	MeOH	1.6	1	100	1.7	6.4	1.71	27 475	n/d
3	Biphasic	Toluene	10.7	24	85	11.6	42.8	0.48	185 375	42^g^
4	CSF (10 mM h^‐1^)	MeOH	2.8	2 + 1^f^	100	1.8	6.7	0.61	29 100	n/d
5	CSF (10 mM h^‐1^)	MeOH	7.0	5 + 1^f^	100	7.2	26.6	1.20	115 000	47
6	CSF (20 mM h^‐1^)	MeOH	8.4	3 + 21^f^	100	8.7	32.1	0.36	138 725	35

a Initial substrate concentration as measured by GC–FID or total substrate concentration added by CSF.

b Conversion and final product concentration calculated from GC–FID, conversion = (1 – ([sub]_final_/[sub]_initial_)) × 100.

c Calculated from the amount of purified TmCHMO‐PTDH in the CFE (M_TmCHMO‐PTDH_ = 100 kg mol^‐1^).

d Experimental space time yield STY_exp_ = [prod]_exp_ / reaction time.

e Turnover number TON = [prod]_exp_ / [TmCHMO‐PTDH].

f (Time of CSF) + (time left to react without substrate feeding).

g 38% from the toluene phase and 5% from the aqueous phase. Reaction conditions: [TmCHMO‐PTDH] = 4 µmol L^‐1^, [NADP^+^] = 250 µmol L^‐1^, 10 % v v^‐1^ (MeOH and ACN) or 33% v v^‐1^ (toluene).

n/d not determined

The process metrics of some batch reactions as well as of the CSF and biphasic systems are shown in Table 2. Although the batch reactions resulted in fast reactions with full substrate conversion and a high STY (1.71 g L^‐1^ h^‐1^ for 10% v v^‐1^ methanol), these reactions afforded low product concentrations (< 2 g L^‐1^) and limited turnover number (TON <28 000) due to the low initial substrate loading. The combination of the low product concentration with low productivity limits the possibility of up‐scaled biocatalyzed oxidations.^27^

The biphasic system in toluene afforded the highest product concentration (11.6 g L^‐1^), biocatalyst yield, and turnover number (TON = 185 375). However, the longer reaction time required for full substrate conversion lowered the productivity of the biphasic system (0.48 g L^‐1^ h^‐1^). Moreover, this strategy requires a more complex product recovery procedure since the substrate and products are present across both phases. A continuous substrate feed at 10 mmol L^‐1^ h^‐1^ with a reaction time of 6 h (Table 2, entry 5) seems to be the best strategy to afford both a higher product concentration (7.2 g L^‐1^) and a higher productivity (1.20 g L^‐1^ h^‐1^) while maintaining a good biocatalyst loading and turnover number (TON = 115 000). Further developments in larger reactors will contribute to improving process metrics and protocol for the BVMO catalyzed oxidation of 3,3,5‐trimethylcyclohexanone.

## CONCLUSIONS

We have demonstrated the viability of the self‐sufficient TmCHMO‐PTDH as a biocatalyst for the synthesis of branched lactones from 3,3,5‐trimethylcyclohexanone as monomer for branched polyesters for speciality materials applications. The effect of the substrate loading, the co‐solvent, the NADP^+^ concentration, and the biocatalyst loading on the specific activity and the conversion were evaluated. Methanol (10% v v^‐1^) was identified as the best co‐solvent for fast reaction rates. A substrate loading of 20 mmol L^‐1^ was found to be the upper limit for good mass balance. Substrate inhibition and limiting product solubility were overcome using two strategies, namely continuous feeding of the substrate (CSF) and a biphasic system with 33% v v^‐1^ toluene, which increased the product concentration as well as the productivity. Further improvements of the biphasic system and product isolation procedures are required to facilitate product recovery and avoid protein denaturation due to the increased interphase. For example, protein denaturation can be limited by controlling the air/liquid interphase via the aeration rate and the stirring speed. Sequential addition of biocatalyst can also contribute to increasing biocatalyst activity, but it will lower the biocatalyst yield. Future work will apply these strategies at larger scale in order to study this system further towards industrial application for lactone monomer synthesis. Overall, this work should contribute to the development of BVMOs as biocatalysts for the synthesis of lactones as polymeric building blocks for polyester production.

## Supporting information


**Figure S1**. Stability of the substrate **1** (black squares) and products **1a** and **1b** (red circles) (analyzed separately) in typical bioconversion reaction conditions without enzyme and with the normalized area from GC‐FID analysis as a function of time.
**Figure S2**. Concentration of substrate (black squares) and product (red circles) for a biocatalyzed reaction with 10 mM substrate and 10% v v^‐1^ acetonitrile. The mass balance (dotted line in blue with y‐axis on the right) was calculated based on the initial substrate concentration: [substrate]_t_ + [product]_t_ / [substrate]_i_
**Figure S3**. Substrate conversion as a function of time × [enzyme] (normalized x‐axis) for increasing TmCHMO‐PTDH concentration. Reactions performed with [substrate] = 10 mM, 10% v v^‐1^ acetonitrile, [NADP^+^] = 250 µM and [phosphite] = 125 mM in KPi buffer (25 mM) at pH 8, 30 °C, airflow of 8 mL min^‐1^.
**Figure S4**. Kinetics of biotransformations with 10% v v^‐1^ co‐solvent a) methanol, b) acetonitrile, c) 1,3‐dioxolane, d) and e) 1,4‐dioxane (left full scale and right zoom‐in), and f) ethanol. Concentrations of substrate (squares with full lines), lactone **1a** (full circles with dotted lines), lactone **1b** (empty circles with dotted lines), and the sum of the product (stars with dotted lines) are given as a function of time. Reactions performed with [substrate] = 10 mM, [TmCHMO‐PTDH] = 4 µM, [NADP^+^] = 250 µM and [phosphite] = 125 mM in KPi buffer (25 mM) at pH 8, 30 °C, 8 mL min^‐1^ airflow.
**Figure S5**. Substrate conversion as a function of time with a) acetonitrile as co‐solvent and [NADP^+^] = 250 µM (black squares) or 100 µM (blue circles); and b) methanol as co‐solvent and [NADP^+^] = 250 µM (black squares) or 50 µM (purple triangles). Reactions performed with [substrate] = 10 mM, 10% v v^‐1^ co‐solvent, [TmCHMO‐PTDH] = 4 µM, and [phosphite] = 125 mM in KPi buffer (25 mM) at pH 8, 30 °C, 8 mL min^‐1^ airflow.
**Figure S6**. Kinetics of biotransformations with 10% v/v BVMO with a) AcCHMO‐PTDH (purple), and b) RhCHMO‐PTDH (blue). Concentrations of substrate (squares with full lines), lactone **1a** (full circles with dotted lines), lactone **1b** (empty circles with dotted lines), and the sum of the product (stars with dotted lines) are given as a function of time. Reactions performed with [substrate] = 10 mM, 10% v v^‐1^ methanol, [NADP^+^] = 250 µM and [phosphite] = 125 mM in KPi buffer (25 mM) at pH 8, 30 °C, 8 mL min^‐1^ airflow.
**Figure S7**. Composition of products (%) as a function of a) substrate concentration, and b) product concentration for 10 mM batch (red triangles), 20 mM batch (black circles), and continuous substrate feeding (CSF) at 10 mM h^‐1^ feeding rate until a total concentration of 50 mM (blue squares). Full symbols indicate the distal lactone **1a** and empty symbols indicate the proximal lactone **1b**. Reactions performed with 10% v/v co‐solvent (acetonitrile for batch reactions and methanol for CSF), 10% v v^‐1^ TmCHMO CFE, and [phosphite] = 125 mM in KPi buffer (25 mM) at pH 8, 30 °C, 8 mL min^‐1^ airflow.
**Figure S8**. Composition of the products for the chemical Baeyer‐Villiger oxidation of 3,3,5‐trimethylcyclohexanone (substrate concentration of 20 mM). Empty circles indicate lactone **1b** and full circles indicate lactone **1a**.
**Figure S9**. Bioconversions with continuous substrate feeding (CSF) of 10 mM h^‐1^ over 5 h with a starting product concentration of 40 mM, with concentration of substrate (black squares) and products (red circles) as a function of time. The black dashed line indicates the substrate feeding rate and the black dotted line indicates the sum of substrate and product concentration. Reactions performed with 10% v v^‐1^ methanol (in the reaction medium from the start of the reaction), [**1a** + **1b**] = 40 mM, [TmCHMO‐PTDH] = 4 µM, and [phosphite] = 125 mM in KPi buffer (25 mM) at pH 8, 30 °C, 8 mL min^‐1^ airflow.
**Figure S10**. Stability of TmCHMO‐PTDH in a toluene/KPi buffer biphasic system with 33% v v^‐1^ toluene at 30 °C (black circles) with remaining activity as a function of time. A blank was measured without toluene (empty circles). The activity was measured by measuring the NADPH consumption by spectrophotometry.
