# Supplementary material for: Application of a thermostable Baeyer–Villiger monooxygenase for the synthesis of branched polyester precursors
Source: J Chem Technol Biotechnol. 2018 Apr 16;93(8):2131–40. doi: 10.1002/jctb.5623 (PMC6055809; doi:10.1002/jctb.5623)
Supplement: Supplementary file 1 — Figure S1. Stability of the substrate 1 (black squares) and products 1a and 1b (red circles) (analyzed separately) in typical bioconversion reaction conditions without enzyme and with the normalized area from GC‐FID analysis as a function of time. Figure S2. Concentration of substrate (black squares) and product (red circles) for a biocatalyzed reaction with 10 mM substrate and 10% v v‐1 acetonitrile. The mass balance (dotted line in blue with y‐axis on the right) was calculated based on the initial substrate concentration: [substrate]t + [product]t / [substrate]i Figure S3. Substrate conversion as a function of time × [enzyme] (normalized x‐axis) for increasing TmCHMO‐PTDH concentration. Reactions performed with [substrate] = 10 mM, 10% v v‐1 acetonitrile, [NADP+] = 250 µM and [phosphite] = 125 mM in KPi buffer (25 mM) at pH 8, 30 °C, airflow of 8 mL min‐1. Figure S4. Kinetics of biotransformations with 10% v v‐1 co‐solvent a) methanol, b) acetonitrile, c) 1,3‐dioxolane, d) and e) 1,4‐dioxane (left full scale and right zoom‐in), and f) ethanol. Concentrations of substrate (squares with full lines), lactone 1a (full circles with dotted lines), lactone 1b (empty circles with dotted lines), and the sum of the product (stars with dotted lines) are given as a function of time. Reactions performed with [substrate] = 10 mM, [TmCHMO‐PTDH] = 4 µM, [NADP+] = 250 µM and [phosphite] = 125 mM in KPi buffer (25 mM) at pH 8, 30 °C, 8 mL min‐1 airflow. Figure S5. Substrate conversion as a function of time with a) acetonitrile as co‐solvent and [NADP+] = 250 µM (black squares) or 100 µM (blue circles); and b) methanol as co‐solvent and [NADP+] = 250 µM (black squares) or 50 µM (purple triangles). Reactions performed with [substrate] = 10 mM, 10% v v‐1 co‐solvent, [TmCHMO‐PTDH] = 4 µM, and [phosphite] = 125 mM in KPi buffer (25 mM) at pH 8, 30 °C, 8 mL min‐1 airflow. Figure S6. Kinetics of biotransformations with 10% v/v BVMO with a) AcCHMO‐PTDH (purple), and b) RhCHMO‐PTDH (blue). [file JCTB-93-2131-s001.docx]

Supplementary information for:

**Application of a thermostable Baeyer-Villiger monooxygenase for the synthesis of branched polyester precursors**

Marie A. F. Delgove, Matthew T. Elford, Katrien V. Bernaerts, Stefaan M. A. De Wildeman

**Table of contents**

**Figure S1**. Stability of the substrate **1** and the products**1a** and **1b** in typical bioconversion reaction conditions.

**Figure S2**. Concentration of substrate and product for a biocatalyzed reaction with 10 mM substrate and 10% v v^-1^acetonitrile.

**Figure S3**. Substrate conversion as a function to time × [enzyme] for increasing enzyme concentration.

**Figure S4.** Kinetics of biotransformations with 10% v v^-1^ co-solvent (methanol, acetonitrile, 1,3-dioxolane, 1,4-dioxane, and ethanol).

**Figure S5**. Substrate conversion as a function of time with a) acetonitrile as co-solvent and [NADP^+^] = 250 μM or 100 μM, and b) methanol as co-solvent and [NADP^+^] = 250 or 50 μM.

**Figure S6**. Kinetics of biotransformations with 10% v v^-1^BVMO with AcCHMO-PTDH and b) RhCHMO-PTDH.

**Figure S7**. Composition of products (%) as a function of a) substrate concentration, and b) product concentration for 10 mM batch, 20 mM batch, and continuous substrate feeding (CSF) at 10 mM h^-1^ until a total concentration of 50 mM.

**Figure S8**. Composition of the products for the chemical Baeyer-Villiger oxidation of 3,3,5-trimethylcyclohexanone (substrate concentration of 20 mM).

**Figure S9**. Bioconversions with continuous substrate feeding (CSF) of 10 mM h^-1^ over 5 h with a starting product concentration of 40 mM.

**Figure S10**. Stability of TmCHMO in a toluene/KPi buffer biphasic system with 33% v v^-1^ toluene at 30 ˚C with remaining activity as a function of time.





**Figure S1**. Stability of the substrate **1** (black squares) and products **1a** and **1b** (red circles) (analyzed separately) in typical bioconversion reaction conditions without enzyme and with the normalized area from GC-FID analysis as a function of time.





**Figure S2**. Concentration of substrate (black squares) and product (red circles) for a biocatalyzed reaction with 10 mM substrate and 10% v v^-1^ acetonitrile. The mass balance (dotted line in blue with y-axis on the right) was calculated based on the initial substrate concentration: [substrate]_t_ + [product]_t_ / [substrate]_i_





**Figure S3**. Substrate conversion as a function of time × [enzyme] (normalized x-axis) for increasing TmCHMO-PTDH concentration. Reactions performed with [substrate] = 10 mM, 10% v v^-1^ acetonitrile, [NADP^+^] = 250 µM and [phosphite] = 125 mM in KPi buffer (25 mM) at pH 8, 30 °C, airflow of 8 mL min^-1^.
















**Figure S4.** Kinetics of biotransformations with 10% v v^-1^ co-solvent a) methanol, b) acetonitrile, c) 1,3-dioxolane, d) and e) 1,4-dioxane (left full scale and right zoom-in), and f) ethanol. Concentrations of substrate (squares with full lines), lactone **1b** (full circles with dotted lines), lactone **1a** (empty circles with dotted lines), and the sum of the product (stars with dotted lines) are given as a function of time. Reactions performed with [substrate] = 10 mM, [TmCHMO-PTDH] = 4 μM, [NADP^+^] = 250 µM and [phosphite] = 125 mM in KPi buffer (25 mM) at pH 8, 30 °C, 8 mL min^-1^ airflow.







**Figure S5**. Substrate conversion as a function of time with a) acetonitrile as co-solvent and [NADP^+^] = 250 μM (black squares) or 100 μM (blue circles); and b) methanol as co-solvent and [NADP^+^] = 250 μM (black squares) or 50 μM (purple triangles). Reactions performed with [substrate] = 10 mM, 10% v v^-1^ co-solvent, [TmCHMO-PTDH] = 4 µM, and [phosphite] = 125 mM in KPi buffer (25 mM) at pH 8, 30 °C, 8 mL min^-1^ airflow.







**Figure S6**. Kinetics of biotransformations with 10% v/v BVMO with a) AcCHMO-PTDH (purple), and b) RhCHMO-PTDH (blue). Concentrations of substrate (squares with full lines), lactone **1b** (full circles with dotted lines), lactone **1a** (empty circles with dotted lines), and the sum of the product (stars with dotted lines) are given as a function of time. Reactions performed with [substrate] = 10 mM, 10% v v^-1^ methanol, [NADP^+^] = 250 µM and [phosphite] = 125 mM in KPi buffer (25 mM) at pH 8, 30 °C, 8 mL min^-1^ airflow.


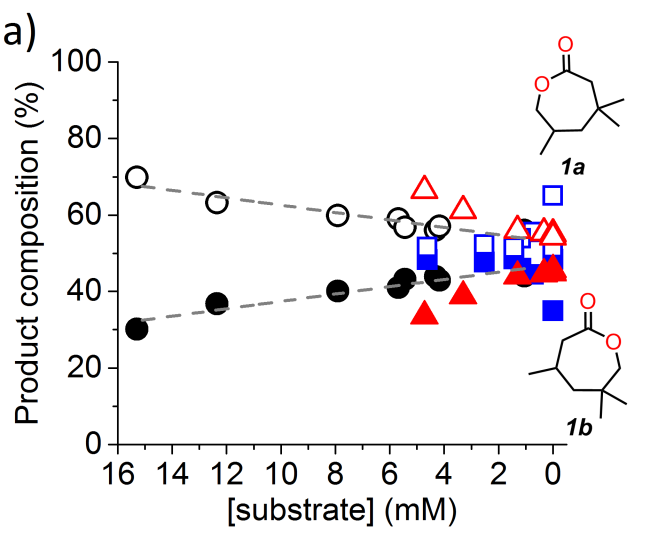

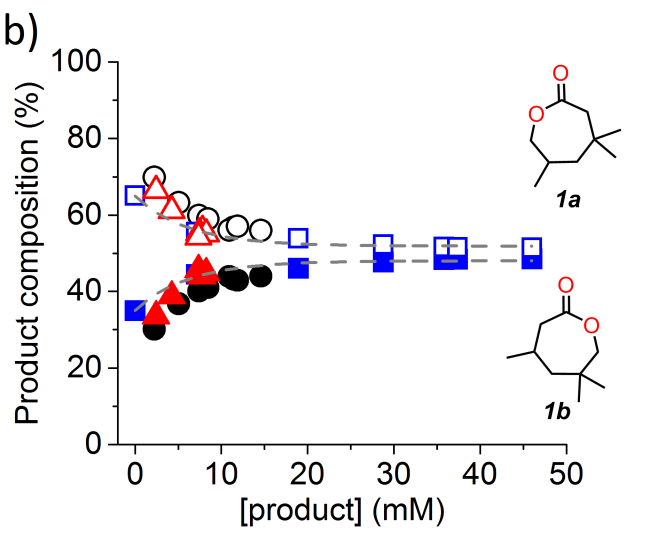


**Figure S7**. Composition of products (%) as a function of a) substrate concentration, and b) product concentration for 10 mM batch (red triangles), 20 mM batch (black circles), and continuous substrate feeding (CSF) at 10 mM h^-1^ feeding rate until a total concentration of 50 mM (blue squares). Empty symbols indicate the distal lactone **1a** and full symbols indicate the proximal lactone **1b**. Reactions performed with 10% v/v co-solvent (acetonitrile for batch reactions and methanol for CSF), 10% v v^-1^ TmCHMO CFE, and [phosphite] = 125 mM in KPi buffer (25 mM) at pH 8, 30 °C, 8 mL min^-1^ airflow.

**
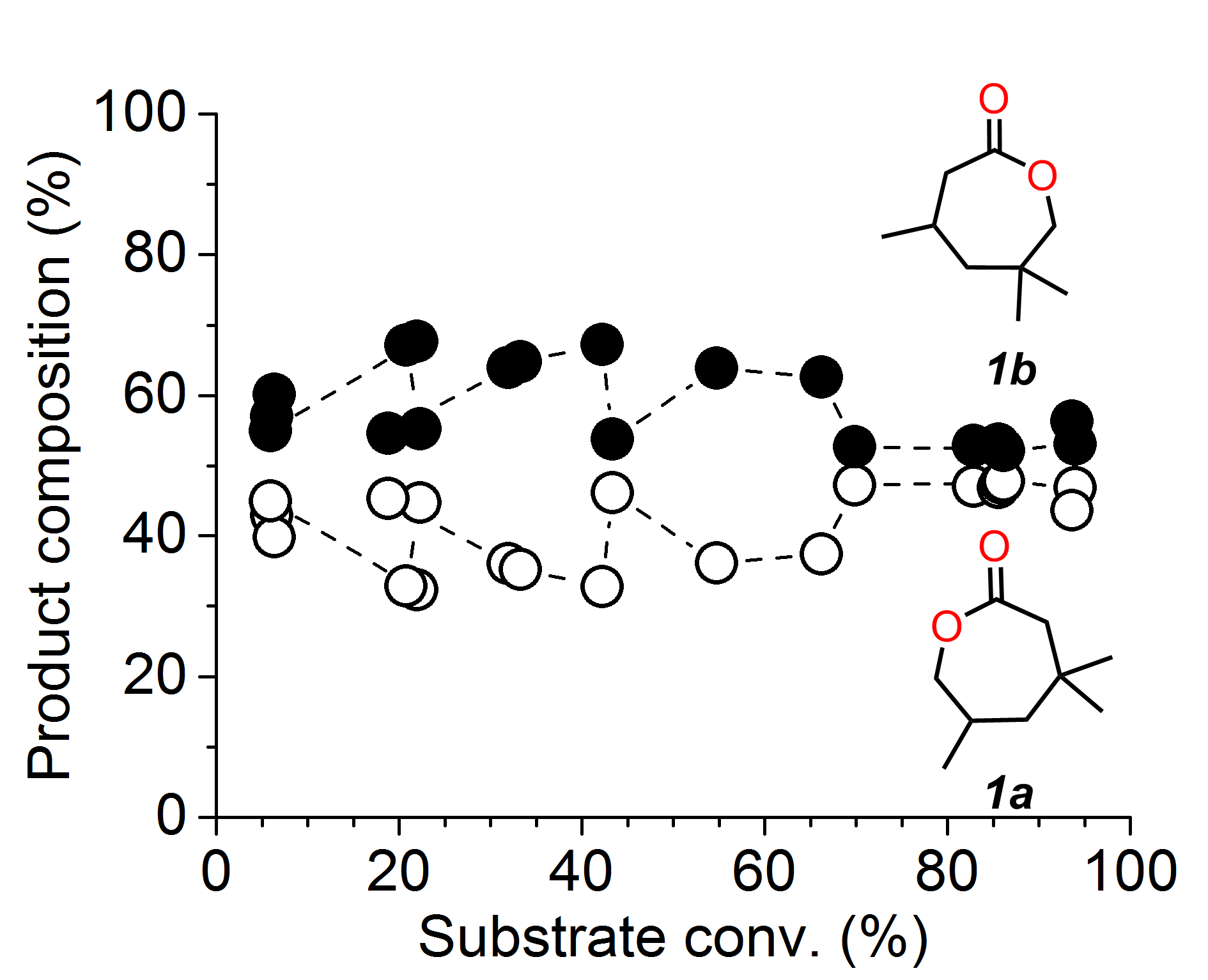
**

**Figure S8**. Composition of the products for the chemical Baeyer-Villiger oxidation of 3,3,5-trimethylcyclohexanone (substrate concentration of 20 mM). Full circles indicate lactone **1b** and empty circles indicate lactone **1a**.





**Figure S9**. Bioconversions with continuous substrate feeding (CSF) of 10 mM h^-1^ over 5 h with a starting product concentration of 40 mM, with concentration of substrate (black squares) and products (red circles) as a function of time. The black dashed line indicates the substrate feeding rate and the black dotted line indicates the sum of substrate and product concentration. Reactions performed with 10% v v^-1^ methanol (in the reaction medium from the start of the reaction), [**1a**+**1b**] = 40 mM, [TmCHMO-PTDH] = 4 µM, and [phosphite] = 125 mM in KPi buffer (25 mM) at pH 8, 30 °C, 8 mL min^-1^ airflow.





**Figure S10**. Stability of TmCHMO-PTDH in a toluene/KPi buffer biphasic system with 33% v v^-1^ toluene at 30 ˚C (black circles) with remaining activity as a function of time. A blank was measured without toluene (empty circles). The activity was measured by measuring the NADPH consumption by spectrophotometry.
